# Secretome from senescent melanoma engages the STAT3 pathway to favor reprogramming of naive melanoma towards a tumor-initiating cell phenotype

**DOI:** 10.18632/oncotarget.1143

**Published:** 2013-08-19

**Authors:** Mickaël Ohanna, Yann Cheli, Caroline Bonet, Vanessa F Bonazzi, Marylin Allegra, Sandy Giuliano, Karine Bille, Philippe Bahadoran, Damien Giacchero, Jean Philippe Lacour, Glen M Boyle, Nicholas F Hayward, Corine Bertolotto, Robert Ballotti

**Affiliations:** ^1^ Inserm U1065, Centre Méditerranéen de Médecine Moléculaire, Equipe 1, Biologie et pathologies des mélanocytes: de la pigmentation cutanée au mélanome. Equipe labellisée Ligue 2013, Nice, F-06204, France; ^2^ Université de Nice Sophia-Antipolis, UFR Médecine, Nice, F-06107, France; ^3^ Centre Hospitalier Universitaire, Service de Dermatologie, Nice, F-06204, France; ^4^ Queensland Institute of Medical Research, 300 Herston Road, Herston, Brisbane 4006, Australia

**Keywords:** melanoma, senescence, secretome, STAT3

## Abstract

Here, we showed that the secretome of senescent melanoma cells drives basal melanoma cells towards a mesenchymal phenotype, with characteristic of stems illustrated by increased level of the prototype genes FN1, SNAIL, OCT4 and NANOG. This molecular reprogramming leads to an increase in the low-MITF and slow-growing cell population endowed with melanoma-initiating cell features. The secretome of senescent melanoma cells induces a panel of 52 genes, involved in cell movement and cell/cell interaction, among which AXL and ALDH1A3 have been implicated in melanoma development. We found that the secretome of senescent melanoma cells activates the STAT3 pathway and STAT3 inhibition prevents secretome effects, including the acquisition of tumorigenic properties. Collectively, the findings provide insights into how the secretome of melanoma cells entering senescence upon chemotherapy treatments increases the tumorigenicity of naïve melanoma cells by inducing, through STAT3 activation, a melanoma-initiating cell phenotype that could favor chemotherapy resistance and relapse.

## INTRODUCTION

Melanoma cells are notoriously known for their high resistance to almost all therapeutic treatments. It was recently proposed that the remarkable phenotypic plasticity of melanoma cells allows for the rapid development of both resistance to chemotherapeutic drugs and invasive properties [1, 2]. At a given time, within a tumor or in culture, not all the melanoma cells have the same capacity to form tumors. A small population is endowed with high tumorigenic potential and has been qualified as Melanoma Initiating Cells (MIC), even though, a reversible phenotypic switch exists between these MIC and their less tumorigenic progeny [3].

Until now no consensual marker, which characterizes the MIC population, has been identified [1, 2, 4-6]. Nevertheless, the most tumorigenic melanoma cells appear to have a poorly differentiated phenotype, with high expression of mesenchymal markers [3, 4, 7-9].

Among the previously described MIC markers, MITF is of particular interest because it is the master transcriptional regulator of melanocyte homeostasis and differentiation [10]. Indeed, we demonstrated that low-MITF expressing cells display MIC properties and increased tumorigenic potential [3]. This population expresses a high level of mesenchymal and stemness markers. The MIC population has been also characterized by their slow growth rate [3, 5], compatible with a stem cell-like phenotype.

Interestingly, the equilibrium between the low-MITF/slow growing, highly tumorigenic and the high-MITF/fast growing, poorly tumorigenic populations can be modified by external stimuli such as a differentiation signal or oxygen level [7]. Therefore, any stimulus that could change this balance, may alter the tumorigenicity of melanoma.

Meanwhile, we showed that melanoma cells could undergo premature senescence upon MITF depletion or upon chemotherapy treatments [11-13]. Senescence can be associated in some circumstances with the production of a secretome composed of several pro-inflammatory factors [14, 15]. In senescent melanoma cells, we demonstrated the existence of such a secretome [16].

While some reports indicated that components of the secretome associated with senescence could reinforce the senescence program [17, 18], other studies demonstrated that it displays deleterious effects, favoring migration, invasion and epithelial-to-mesenchymal transition [2, 19]. In agreement with these results, we found that exposure of naive, non-tumorigenic 501mel melanoma cells with the secretome of senescent melanoma cells (SSMC), rendered them highly tumorigenic [16]. These observations raise the possibility that chemotherapy treatments may have deleterious effects by promoting the tumorigenic potential of cryptic, residual melanoma cells, not affected by the treatment, and thus contribute to the recurrence of the disease.

Here, we provide evidence that melanoma cells of different genetic backgrounds exhibit changes in the expression of *EMT*-related and stemness genes after exposure to the SSMC. Induction of the mesenchymal phenotype was accompanied by an increase in the low-MITF, slow growing population. All these features indicate that SSMC facilitates the switch toward a melanoma-initiating cell phenotype and favors thereby an increase in tumorigenic potential.

Our findings also point out, both in vitro and in vivo, the key role of the STAT3 signaling cascade in acquisition of the stemness and mesenchymal phenotype as well as the melanoma-initiating properties mediated by SSMC. Targeting the STAT3 pathway might help to circumvent the detrimental effects of the SSMC and to strengthen the efficiency of anti-melanoma therapies.

## RESULTS

### The secretome of senescent melanoma cells increases the melanoma initiating cell population

**Figure 1:**
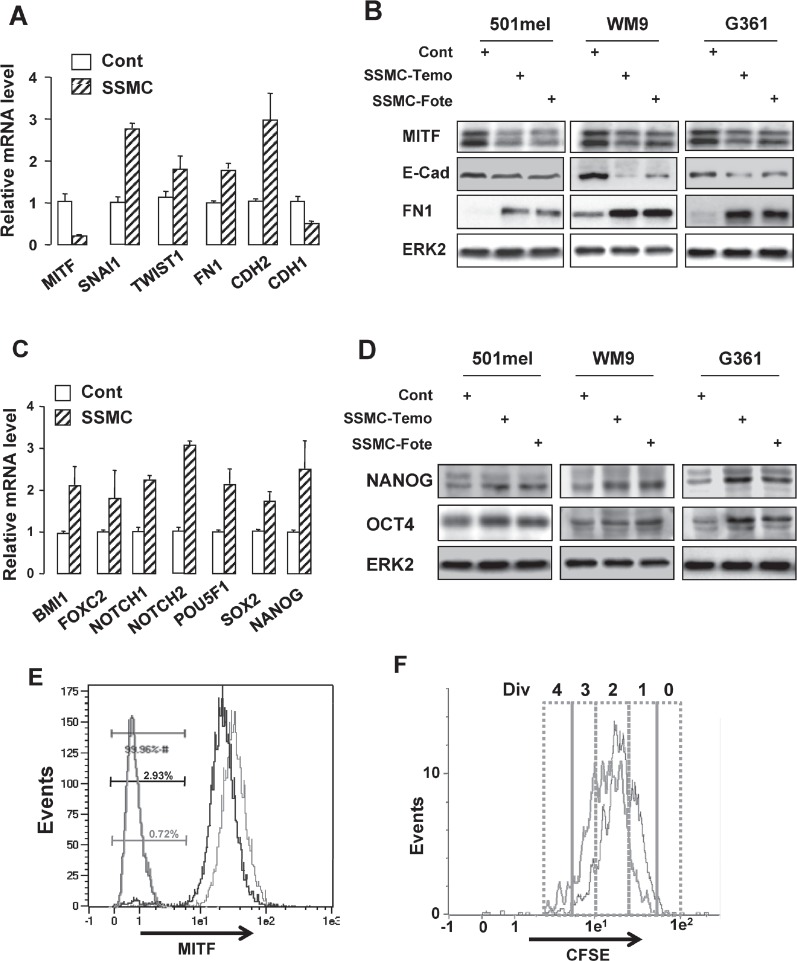
The secretome of senescent melanoma cells enhances the melanoma initiating cell population

We have shown that exposure of naïve melanoma cells to the SSMC increases their motility and tumorigenic properties [16]. To explore the mechanism involved in these processes, we first analyzed the effect of this secretome on the expression of mesenchymal markers. QRT-PCR experiments showed that naïve 501mel melanoma cells exposed to the secretome of melanoma cells rendered senescent by MITF silencing exhibited an increased level of SNAIL1, TWIST1, Fibronectin1, N-Cadherin (CDH2) mRNAs whereas MITF and E-Cadherin (CDH1) transcripts were markedly decreased (Fig. 1A).

(A and C) RNAs were harvested from 501mel cells exposed to the secretome of control melanoma cells transfected with control siRNA (Cont) or of melanoma cells rendered senescent by MITF silencing (SSMC) for 48 hrs and were assayed by qRT-PCR for transcripts indicated on the figure. Transcript levels are represented relative to those found in control cells as mean +SD. (B and D) Lysates from 501mel human melanoma cells exposed 48 hrs to the secretome of control melanoma cells (Cont) or of melanoma cells rendered senescent by exposure to temozolomide, 900 nM (SSMC-Temo) or fotemustine, 40 nM (SSMC-Fote), were analyzed by western blotting with MITF, ECAD, FN1, NANOG and OCT4 antibodies. ERK2 was used as loading control. (E) Cells freshly isolated from human biopsies (Patient#1) exposed to the secretome of control melanoma cells (red line) or SSMC (black line) for 48 hrs, were analyzed for MITF content by flow cytometry. MITF intensity was plotted on abscissa and side scatter (SSC) on ordinates. An isotype-matched control antibody (green line) has been used as a negative control. (F) 501mel cells were labeled with 2 μM CFSE according to the manufacturer's protocol and grown in the conditioned media of secretome of control melanoma cells (red line) or SSMC (black line) for 72 hrs. Cells were next analyzed by flow cytometry for CFSE intensity. An estimation of the number of divisions (div) was determined according to CFSE intensity (0-4).

Similar effects were observed when senescence was induced by chemotherapy drugs used in first line treatment of melanoma. Indeed, three different melanoma cell lines exposed to the secretome collected from melanoma cells rendered senescent by temozolomide or fotemustine displayed increased expression of Fibronectin1 while exhibiting a decrease in E-Cadherin and MITF protein level (Fig. 1B, Supplementary Fig. S1A).

The reduction in MITF and the gain of the mesenchymal phenotype were associated with an increase in stemness markers [3]. Therefore, we studied the effect of the secretome of melanoma cells rendered senescent by MITF silencing on these markers. QRT-PCR experiments showed that SSMC led to an increase in expression of several stemness markers including BMI1, FOXC2, NOTCH1/2, POU5F1 (OCT4), SOX2 and NANOG (Fig. 1C). We also observed increased expression of OCT4 and NANOG when naïve cells were exposed to the secretome of melanoma cells rendered senescent by the chemotherapy drugs (Fig. 1D).

These observations indicate that SSMC favors the acquisition of mesenchymal and stemness phenotypes by naive melanoma cells. These features are hallmarks of tumor-initiating cells, which were also characterized in melanoma by a low level of MITF expression and a transient slow growing rate [3, 5].

Flow cytometry analysis showed that exposure to SSMC increased the low-MITF population (70% to 300%) in 501mel, G361, MeWo and WM9 cell lines, which have different BRAF mutational status (Supplementary Fig. S1B), and in melanoma cells isolated from patient skin metastasis expressing wild type BRAF, in which SSMC (black line) led to a 4-fold increase in the percentage of the low-MITF population (0.72 to 2.93%) (Fig. 1E). We also observed that SSMC decreased the growth of different melanoma cell lines, as illustrated by cell counting (Supplementary Fig. S1C), but did not trigger cell death in 501mel and in other melanoma cells, in contrast to staurosporine used as a positive control (Supplementary Fig. S1D). Then, using carboxyfluorescein diacetate succinimidyl ester (CFSE), a vital dye whose fluorescence intensity decreases by half at each cell division [20], we studied the division potential of melanoma cells exposed to SSMC. After CFSE loading, 501mel cells were cultured for 4 days with or without SSMC and analyzed by FACS (Fig. 1F). Under control conditions, most (80%) of 501mel cells underwent 2 to 3 divisions and a small percentage (8%) of cells underwent only 0/1 division (red line). When naïve melanoma cells were incubated with SSMC (black line) the percentage of the slow-growing cells (0/1 division) increased to 30% (Supplementary Table S1).

Therefore, naive melanoma cells, exposed to the conditioned medium of melanoma cells entering a program of senescence, experience a phenotypic switch that recapitulates the hallmarks of melanoma-initiating cells, i.e. increase in mesenchymal and stemness markers, augmentation of a low-MITF and slow-growing population.

### Activation of the STAT3 signaling pathway by the secretome of melanoma cells undergoing senescence

To go deeper into the molecular mechanisms by which the senescent conditioned medium drives the reprogramming of melanoma cells, we assessed the effect of the SSMC on several signaling pathways that play critical roles in melanoma.

**Figure 2:**
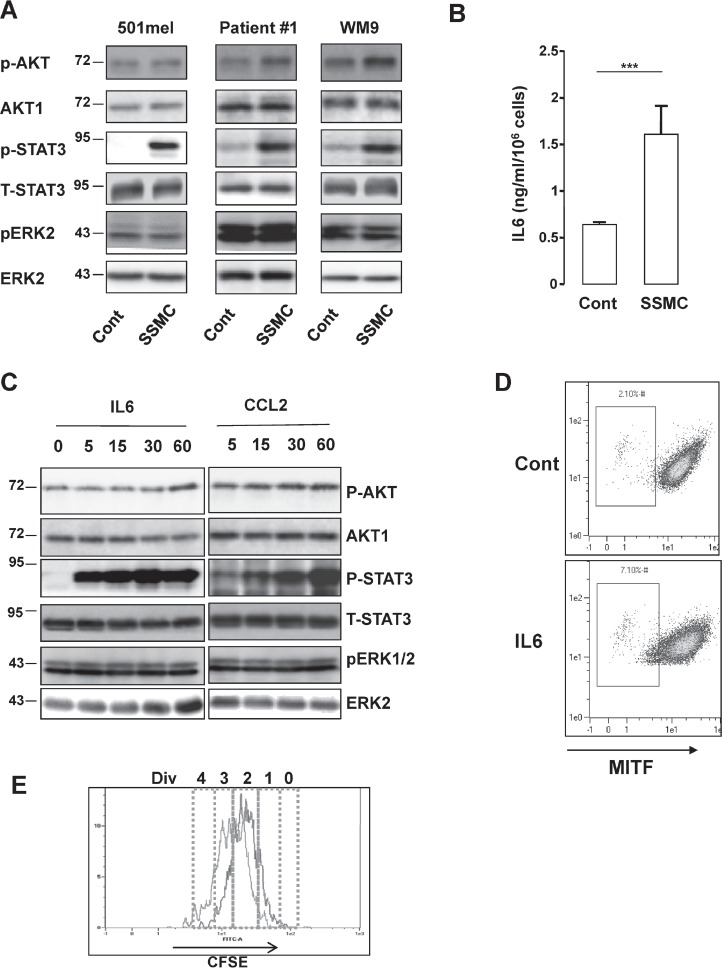
Activation of the STAT3 signaling pathway by the secretome of senescent melanoma cells

Western blot with phospho-specific antibodies directed against activated ERK, AKT or STAT3 indicated that melanoma cells of different genetic backgrounds exposed to SSMC displayed a strong activation of STAT3. No consistent activation of ERK and a weak AKT phosphorylation could be observed under the same conditions (Fig. 2A).

(A) Human 501mel and WM9 melanoma cells lines and cells freshly isolated from human biopsies (Patient#1) were exposed 1 hr to the secretome of control (Cont) or senescent (SSMC) melanoma cells. Lysates were analyzed by western blot for the indicated antibodies. (B) ELISA test of IL6 level in control or senescent secretome. Data, represented as mean +SD, are significantly different ***p<0.001. (C) 501mel melanoma cells were treated with recombinant IL6 (20 ng/ml) or CCL2 (200 ng/ml) for the time indicated. Lysates were analyzed by western blot for the indicated antibodies. (D) 501mel cells in control condition or incubated with IL6 (20 ng/ml) for 48 hrs were analyzed for MITF content by flow cytometry. MITF intensity was plotted on abscissa and side scatter (SSC) on ordinates. (E) 501mel cells were left in control condition (red line) or incubated with IL6, 20 ng/ml for 72 hrs (black line). Cells were labeled with 2 μM CFSE according to the manufacturer's protocol and next they were analyzed by flow cytometry for CFSE intensity. An estimation of the number of divisions (div) was determined according to CFSE intensity (0-4).

The STAT3 signaling pathway has been shown to be involved in the control of stemness and is activated by numerous cytokines present in the secretome of senescent melanoma, including CCL2 [16]. Here, we extended these results and showed by ELISA that IL6 was also markedly increased in the SSMC (Fig. 2B).

Time course experiments showed that both CCL2 and IL6 induced a dramatic increase in STAT3 activation, as illustrated by its tyr705-phosphorylation (Fig. 2C and supplementary Fig. S2). IL6 achieved a very rapid and robust STAT3 phosphorylation within 5 min, while the effect of CCL2 was more progressive and reached its maximum at 60 min. Western blots also revealed that CCL2 and IL6 triggered no or very weak stimulation of AKT and ERK phosphorylation (Fig. 2C). Focusing on IL6, FACS analysis revealed that this cytokine increased the low-MITF population from 2 to 7% (Fig. 2D). IL6 also favored the apparition of the slow-growing population by increasing the percentage of cells that underwent 0/1 division (5.65 to 18.3%) (Fig. 2E and supplementary Table S2). Therefore, IL6 is able to mimic the effects of the SSMC to favor the melanoma-initiating cell phenotype.

### STAT3 activation mediates the acquisition of the stemness phenotype elicited by SSMC or by IL6

**Figure 3:**
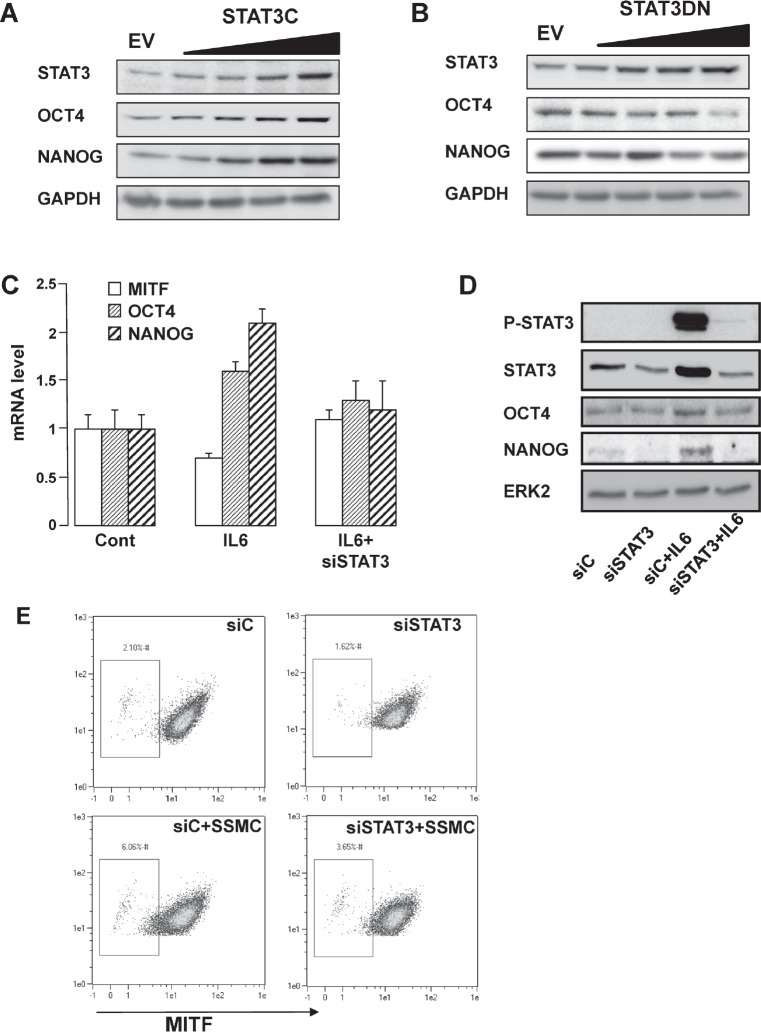
STAT3 activation mediates the acquisition of the stemness phenotype in melanoma cells

Overexpression of a constitutively *active mutant* of STAT3 (STAT3C) mimicked the effect of the SSMC or of IL6 on the stemness markers OCT4 and NANOG (Fig. 3A). Conversely, inhibition of STAT3 activity by overexpressing a dominant negative form (STAT3DN) promoted a decrease in OCT4 and NANOG protein level (Fig. 3B). Therefore, STAT3 activity appears to parallel the expression of NANOG and OCT4.

(A-B) 501mel cells were infected with increasing concentrations of lentivirus encoding either the parental empty vector (EV) or vectors encoding either a constitutive active form (STAT3C) or a dominant negative form (STAT3DN) of STAT3. Western blotting assays were performed with the indicated antibodies. (C) QRT-PCR experiments on RNAs prepared from 501mel cells left in control condition, exposed to IL6 (20 ng/ml) or transfected with a STAT3 siRNA cells and treated with IL6. Transcript levels are represented relative to those found in control cells as mean +SD. (D) Western blotting assays of 501mel cells transfected with a control (siC) or STAT3 siRNA left untreated or treated with recombinant IL6. (E) FACS analysis of MITF intensity of cells treated as in D. MITF intensity was plotted on abscissa and side scatter (SSC) on ordinates.

In agreement with the above observations, IL6 enhanced NANOG and OCT4 expression while it reduced the amount of MITF, at the mRNA and protein levels (Fig. 3C-D). STAT3 inhibition by siRNA prevented the effects of IL6 on OCT4 and NANOG expression. Additionally, flow-cytometry analysis revealed that the increase in the low-MITF population elicited by the SSMC (2% vs 6%) was inhibited by about 50% upon STAT3 silencing (6% vs 3.65%) (Fig. 3E). Hence, STAT3 activation is required for the acquisition of the melanoma-initiating cell properties induced by the SSMC or by IL6.

Next, we sought to identify the whole transcriptome modifications triggered by the SSMC. Towards this aim, two melanoma cell lines (501mel and WM9) and melanoma cells freshly isolated from a patient were transfected with STAT3 siRNA, or scrambled siRNA, then exposed or not to the SSMC. As shown by western blot in the three melanoma cell types, STAT3 siRNA efficiently reduced STAT3 expression and the SSMC activated STAT3 compared to the control conditioned medium (Supplementary Fig. S3). Furthermore, the reduced MITF expression mediated by SSMC was clearly abrogated in WM9 and patient#1 cells when STAT3 was knocked down. Expression array analysis highlighted a signature of 52 genes upregulated by the SSMC in the three melanoma cell types (Table 1). The regulation of all these genes was prevented by STAT3 inhibition, strengthening the key role of STAT3 in this process. Noteworthy, Fibronectin1 (FN1), OCT4, NANOG and MITF do not belong to the 52-gene list. However, a careful analysis of the data revealed that most of these genes were below background detection limits or did not pass the statistical threshold.

**Table 1:** List of the 52 genes regulated by the exposure to SSMC for 24h, in 501Mel, WM9 melanoma cells from patient#1 Log ratio threshold>1. None of these genes is regulated by SSMC in cells transfected with siRNA STAT3.

Symbol	Log Ratio	p-value	Symbol	Log Ratio	p-value	Symbol	Log Ratio	p-value
**DKK3**	5.489	6.39E-03	**TMEM171**	1.698	1.65E-02	**CHST3**	1.32	1.46E-03
**SCRG1**	4.378	1.66E-02	**CCL2**	1.686	2.00E-02	**ARMCX2**	1.27	1.20E-02
**TGFBI**	4.188	7.57E-04	**KLF9**	1.674	2.55E-03	**SPOCK1**	1.247	1.07E-02
**TIMP4**	3.733	8.66E-03	**TMEM47**	1.66	4.74E-03	**MYOF**	1.234	1.21E-02
**LOXL4**	3.679	5.74E-03	**KCNMA1**	1.569	2.50E-03	**STK32B**	1.224	3.22E-03
**ALDH1A3**	3.495	4.17E-03	**A2M**	1.563	8.96E-04	**SYNM**	1.217	2.56E-03
**ST8SIA5**	2.972	7.24E-03	**CDH13**	1.533	2.08E-03	**CA12**	1.187	2.83E-03
**TIMP3**	2.796	2.36E-03	**PDGFRA**	1.514	1.08E-03	**TNFRSF6B**	1.184	1.47E-02
**PXDN**	2.767	1.13E-03	**IL1RAPL1**	1.502	1.00E-02	**LAMA4**	1.15	2.02E-03
**SFRP1**	2.45	1.99E-02	**PASD1**	1.448	1.75E-02	**PRSS23**	1.145	1.20E-02
**HTATIP2**	2.225	6.54E-03	**SDC4**	1.429	3.78E-03	**GBP2**	1.087	3.37E-03
**PCOLCE**	2.134	1.05E-02	**SLFN11**	1.348	1.63E-03	**HS3ST3A1**	1.083	5.72E-03
**BGN**	2.126	1.20E-02	**VAT1L**	1.323	8.13E-03	**TNS3**	1.064	5.96E-03
**1 LIB**	2.096	1.55E-02	**CHST3**	1.32	1.46E-03	**ITIH6**	1.059	4.02E-03
**AXL**	2.072	4.65E-03	**PDGFRA**	1.514	1.08E-03	**SRPX**	1.057	1.29E-03
**PMEPA1**	1.889	4.80E-03	**IL1RAPL1**	1.502	1.00E-02	**EFEMP2**	1.048	1.36E-02
**FAM129A**	1.815	2.44E-04	**PASD1**	1.448	1.75E-02	**CRMP1**	1.028	3.22E-03
**COL8A1**	1.79	3.02E-03	**SDC4**	1.429	3.78E-03	**GNG2**	1.025	4.23E-03
**RGL1**	1.73	6.13E-03	**SLFN11**	1.348	1.63E-03	**SLC38A1**	1.024	5.70E-03
**EIF1AY**	1.724	1.30E-02	**VAT1L**	1.323	8.13E-03			

Nevertheless, it should be noted that several known MITF target genes such as MLANA, RAB27a or SLC24A5 were weakly but significantly downregulated in all three melanoma cells, in accordance with the observations of a reduced MITF expression (Supplementary Table S3).

Re-assessment of the gene expression profile by qRT-PCR confirmed the markedly increased mRNA levels of AXL, ALDH1A3, CCL2, TNC, THBS2, DKK3 and TGFBI identified by the microarray experiments, also to a lesser extent that of FN1, OCT4, NANOG and the reduction of MITF (Supplementary Fig. S4A). Ingenuity Pathway Analysis indicated that the major molecular and cellular functions associated with the genes enhanced by the SSMC were Cell movement, Cell-To-Cell Signaling and Interaction and Cellular Growth and Proliferation (Supplementary Fig. S4B). Therefore, the SSMC induces a molecular reprogramming of melanoma cells toward a more motile and tumorigenic phenotype, mainly through STAT3 activation.

### STAT3 activation mediates the acquisition of tumorigenic phenotype elicited by SSMC or by IL6 and is required for melanoma growth in vivo

**Figure 4:**
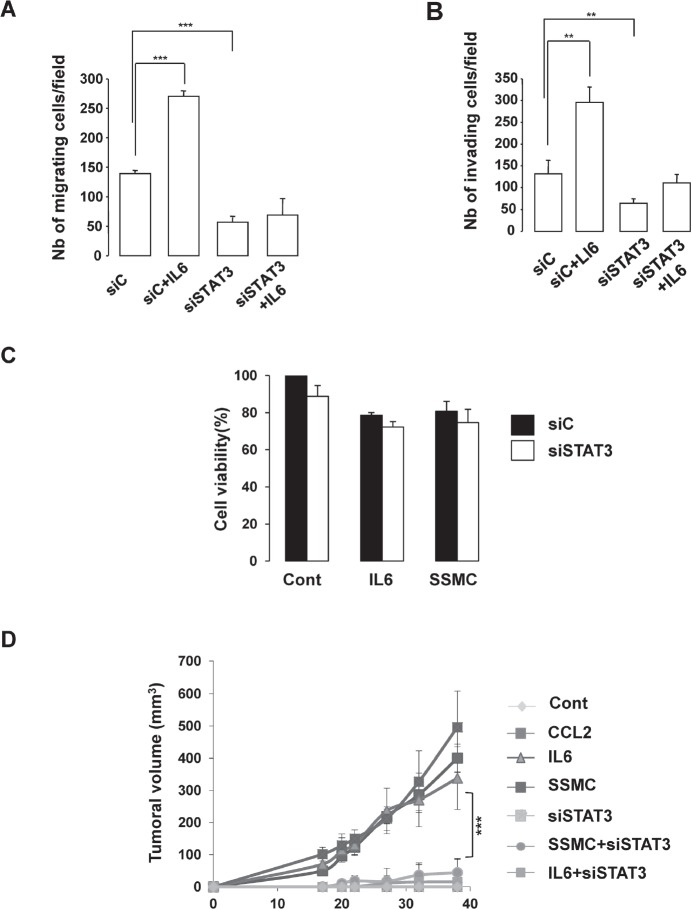
STAT3 activation is required for the acquisition of the tumorigenic phenotype of melanoma cells

Finally, we evaluated the effect of STAT3 silencing on the biological behavior of melanoma cells. In vitro experiments using Boyden chambers demonstrated that STAT3 siRNA decreased both basal and IL6 induced-migration and matrigel dependent invasion (Fig. 4A-B). However, STAT3 siRNA did not affect significantly cell growth or viability (Fig. 4C). To determine the effect of the secretome in vivo, we used 501mel human melanoma cells that do not grow as xenografts in athymic nude mice. Cells exposed in vitro to secretome of control melanoma cells or cells transfected with STAT3 siRNA before transplantation did not form tumors. However, 501mel exposed to SSMC, CCL2 or IL6 gave rise to tumors (Fig. 4D). Importantly, the pro-tumorigenic effects of SSMC or of IL6 were completely abrogated when cells were knocked down for STAT3, thereby demonstrating the key role of STAT3 activation in the acquisition of the tumorigenic potential by melanoma cells.

(A-B) 501mel cells, transfected with a control (siC) or STAT3 siRNA, were left untreated or treated with recombinant IL6 (20 ng/ml) for 48 hrs and next were seeded on the filter of the upper compartment non coated (migration) or coated (invasion) with matrigel. DMEM with 7% serum were added to the lower compartment of Boyden chambers. Cells that had migrated to the underside of the upper compartment were stained 24 hrs later with crystal violet and the number of nuclei was counted using NIH-*imageJ* analysis software. Values represent mean +SD of three independent experiments, significantly different from the control ***p<0.001 and ** p<0.01. (C) 501mel cells, transfected with control (siC) or STAT3 siRNA were exposed to secretome of control melanoma cells (C), IL6 (20 ng/ml) or to the SSMC for 48 hrs and then subject to a cell viability assay. (D) 501mel melanoma cells treated as in (C) were subcutaneously injected in athymic nude mice. Values are expressed as mean ±SEM.

## DISCUSSION

Heterogeneity and plasticity are the two biological phenomena that might be responsible for the remarkable resistance of melanoma to the current therapeutic armamentarium. Both phenomena can be explained by the concept of melanoma initiating cells, which are thought to derive from the phenotypic switch of more differentiated melanoma cells [21]. It has been shown that stimuli such as hedgehog [22] or hypoxia [7] can increase the MIC population, favoring thereby tumorigenicity. We previously reported that chemotherapy drugs, used in melanoma treatment, entail a senescence-like phenotype in melanoma cells that is associated with the production of an inflammatory secretome (SSMC) endowed with pro-tumorigenic properties [16]. Therefore, we hypothesized that SSMC might favor melanoma tumorigenicity also by increasing MIC population.

Here, we show that the SSMC and IL6, one of its components, enhance the expression of mesenchymal and stemness markers, that pairs with an increase in the MIC population defined by a slow-growing rate and a low-MITF expression. These observations are in agreement, with several reports demonstrating that IL6, which belongs to the LIF family, favors the transition toward cancer stem cells in breast and prostate cancers [23], and increases mesenchymal transition of melanoma cells and melanoma tumor development [24]. Further, melanoma development is delayed in IL6 deficient mice [25].

**Figure 5:**
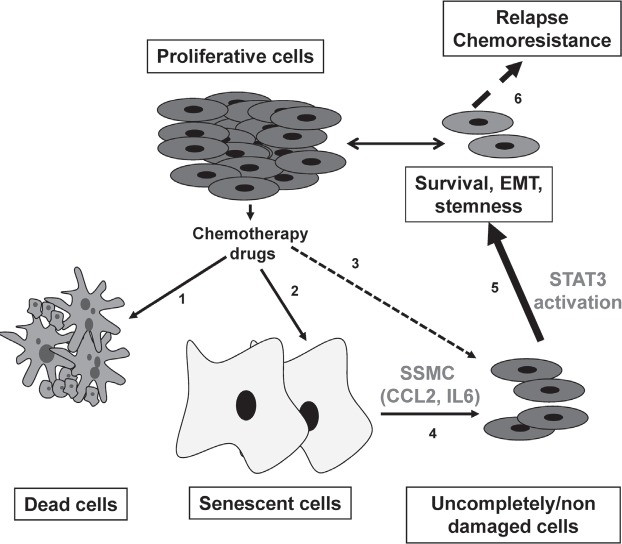
Model for melanoma chemoresistance

Study of the signaling pathways engaged by exposure to the SSMC showed no activation of ERK, a weak activation of AKT and a robust activation of STAT3. The Signal Transducer and Activator of Transcription-3 (STAT3) is a member of the STAT family that relays extracellular signals initiated by cytokines and growth factors from the cytoplasm to the nucleus [26]. The constitutive activation of STAT3 is frequently detected in human cancer, including melanoma, and is associated with poor clinicopathological features and prognosis [27, 28]. Recently, STAT3 activation has been associated with tumor initiating cell phenotype of liver, colon and glial cancer cells [29-31]. Further, the activation of STAT3 has been also involved in the acquisition/maintenance of the pluripotency by controlling the expression of KLF4 and NANOG [32]. Our results demonstrate that CCL2 and IL6, which are present in the SSMC, also promote an activation of STAT3 and that STAT3 silencing prevents the acquisition of the mesenchymal, stemness and melanoma-initiating phenotypes. Collectively these observations indicate that STAT3 is a key player in the transition between melanoma initiating cells and their more differentiated progeny (Fig. 5).

Chemotherapy treatment triggers cell death (1) and senescence (2) while sparing some cancer cells due to microenvironment or limited vascularisation (3). Senescent cells produce an inflammatory secretome composed of CCL2 and IL6 (4) that could enhance, through STAT3 activation, the survival and the acquisition of more mesenchymal and stemness properties of spared cancer cells (5), that in turn causes chemoresistance and relapses (6).

Therefore, we sought to identify the repertoire of genes regulated by the SSMC that are under the control of STAT3. Transcriptomic studies and Ingenuity Pathway Analysis revealed that SSMC increases the expression of a set of genes related to Cell movement, Cell-To-Cell Signaling and Interaction and Cellular Growth and Proliferation. Among these genes, 15 are both up-regulated in melanoma cell lines with invasive phenotype [33] and down-regulated in A375 melanoma cells over-expressing MITF [34] (Supplementary Table S4). Therefore, the transcriptomic profile matches perfectly with the acquisition of a more invasive phenotype and melanoma-initiating cell properties. The inhibition of STAT3 by siRNA completely prevented the regulation of all these genes, reinforcing the notion that STAT3 is a critical mediator of melanoma-initiating features and melanoma aggressiveness.

Among the direct or indirect STAT3 targets, upregulated by SSMC; ALDH1A3 was recently associated with melanoma-initiating properties [22, 35], even though not all reports agree with this hypothesis [36]; tenascin (TNC), an extracellular matrix protein is an important component of stem cell niches [37] that favors evasion of tumor cells, including melanoma [38], from conventional therapy and might therefore participate in the acquisition of the MIC phenotype. Finally, the upregulation of AXL by SSMC is of particular interest, because AXL belongs to the TAM (Tyro3, AXL, MER) family of receptor tyrosine kinase involved in various cancers, including melanoma [39]. AXL was thought to play a key role in the acquisition of the resistance to EGFR inhibitor in lung cancer [40]. In melanoma, AXL is activated and preferentially expressed in melanoma lacking MITF [41]. In agreement with our data, AXL favors melanoma migration.

How STAT3 regulates these genes remains to be elucidated. Of course STAT3 can directly bind to the promoter of the up-regulated genes to control their expression. However, the transient activation of STAT3 during in vitro exposure of melanoma cells to SSMC is sufficient to increase the tumorigenic potential and favors the subsequent tumor development, indicating that the consequences of STAT3 activation continue for several weeks and cell divisions, to allow tumor development. This effect persisting despite the cessation of the initial stimuli might also be the consequence of an epigenetic regulation. This hypothesis is in agreement with a recent report demonstrating that STAT3 signaling promotes somatic cell reprogramming by epigenetic regulation [42] and may provide a link between the inflammatory response, epigenetic remodeling and cancer development.

Finally, the inhibition of STAT3 expression by siRNA abrogates the acquisition of the tumorigenic properties evoked by the in vitro exposure of melanoma cells to SSMC and completely prevents the development of xenografts. This observation strengthens the pivotal role of STAT3 in melanoma development and provides a rational for evaluation of STAT3 inhibitors in melanoma treatment, alone or in combination with existing therapies.

## METHODS

### Cell cultures

501mel, G361, WM9, MeWo and patient human melanoma cells (Patient#1) were grown in DMEM supplemented with 7% FBS at 37°C in a humidified atmosphere containing 5%CO2. Naïve melanoma cells were defined as melanoma cells maintained in their basal culture medium until they were exposed to the secretome of melanoma cells rendered senescent.

Cell isolation from human biopsies was previously described [11].

### Antibodies and Reagents

Anti-MITF (Ab80651) and anti-Nanog antibodies were Abcam, anti-ERK2 (sc-1647 clone D-2) and anti-Oct4 antibodies were from Santa Cruz biotechnology. STAT3 (#9132), pTyr705STAT3 (#9131), pS473AKT (#9271), pThr202/Tyr204ERK1/2 (#9101) antibodies were from Cell Signaling Technology. Anti-fibronectin (#610077BD) and anti-E-cadherin (#610404BD) antibodies were from Transduction laboratories. Horseradish peroxidase-conjugated anti-rabbit or anti-mouse antibodies were from Dakopatts (Glostrup, Denmark). *Recombinant human CCL2 and IL6* were from R&D Systems. Secondary alexa488 antibody, propidium iodide, DAPI, CellTrace CFSE and XTT cell proliferation kit were from Invitrogen.

### Transient transfection of siRNA

Briefly, a single pulse of 50 nM of siRNA was administrated to the cells at 50% confluency by transfection with 5 μl lipofectamine^TM^ RNAiMAX in opti-MEM medium (Invitrogen, San Diego, CA, USA). Control (siC) and MITF (siMi) siRNAs were previously described [43]. STAT3 siRNA were purchased at Dharmacon Inc.

### Migration and Invasion Assays

Cell migration and invasion were assessed using a modified Boyden chamber assay with 8-μm pore filter inserts for 24-well plates (BD Bioscience). 501mel cells were *seeded on the upper chamber* of uncoated or matrigel-coated filters and DMEM 7% SVF placed into the lower chamber. Twenty-four hours later, *cells* adherent to the *underside of the filters* were fixed with 4% PFA, stained with 0.4% crystal violet and five random fields at x20 magnification were counted. Results represent the average of triplicate samples from three independent experiments.

### Western blot assays

Western blots were carried out as previously described [44]. Briefly, cell lysates (30 μg) were separated by SDS-PAGE, transferred on to a PVDF membrane and then exposed to the appropriate primary and HRP-linked secondary antibodies. Proteins were visualized with the ECL system (Amersham). The western blots shown are representative of at least 3 independent experiments.

### mRNA preparation, Real-time/quantitative PCR

mRNA isolation was performed with Trizol (Invitrogen), according to standard procedure. QRT-PCR was carried out with SYBR® Green I and Multiscribe Reverse Transcriptase (Promega) and monitored by an ABI Prism 7900 Sequence Detection System (Applied Biosystems, Foster City, CA). Detection of SB34 gene was used to normalize the results. Primer sequences for each cDNA were designed using either Primer Express Software (Applied Biosystems) or qPrimer depot (http://primerdepot.nci.nih.gov) and are available upon request.

### ELISA

IL6 level in the secretome of 501mel melanoma cell lines was quantified by ELISA (R&D Systems). Results from two independent experiments were normalized to cell number and expressed as ng/ml/10^6^ cells.

### Tumor models

Animal experiments were carried out in accordance with French law and were approved by a local institutional ethical committee. Animals were maintained in a temperature-controlled facility (22°C) on a 12-hour light/dark cycle and were given free access to food (standard laboratory chow diet from UAR, Epinay-S/Orge, France).

Mice were randomly divided into 3 groups of 6 mice. 501mel melanoma cells were transfected with control (siC) or STAT3 (siSTAT3) siRNA and were exposed in vitro to the secretome of senescent melanoma cells (SSMC) or to recombinant CCL2 (200 ng/ml) or IL6 (20 ng/ml) for 48 hrs, washed twice with phosphate-buffered saline and then inoculated subcutaneously (4×10^6^ cells/mouse) into 6-week-old female immune-deficient Athymic Nude FOXN1^*nu*^ mice (Harlan Laboratory). The growth tumor curves were determined by measuring the tumor volume using the equation V=(L × W^2^)/2. Mice were killed by CO_2_ inhalation and tumors were taken.

### CFSE labeling and FACS analysis

For the in vitro CFSE assay, cells were labeled with 2 μmol/l of CFSE according to the manufacturer's protocol (Invitrogen), then plated and treated with the secretome of control (SCMC) or senescent melanoma cells (SSMC) or recombinant IL6 (20 ng/ml). Cells were detached and analyzed by flow cytometry using MACSQuant (Miltenyi biotech). Cells were exposed to control (Cont) or senescent secretome (SSMC). Seventy-two hours later, cells were detached in phosphate-buffered saline/EDTA 2 mM and stained with DAPI to exclude dead cells. Fluorescence was measured by using the FL1/FL2 channels of a MACSQuant. Data were analyzed with MACSQuant software (Miltenyi biotech).

### Cell viability test

Cell viability was assessed using the cell proliferation kit II (XTT; Roche Molecular Biochemicals, Indianapolis, IN) according to the manufacturer's protocol. Cell viability, measured at 490 nm, is expressed as the percentage of the value of control cells.

### Statistical Analysis

Data are presented as averages ±SD and were analyzed by student t-test using Microsoft Excel software. A p value of 0.05 (*p<0.05) or less (**p<0.01 and ***p<0.001) was interpreted as indicating statistical significance when comparing experimental and control groups.

### Expression Profiling and Analysis

Biotinylated cRNA was prepared with the Illumina TotalPrep RNA Amplification Kit (Ambion, Austin, TX, USA). Labelled cRNA was hybridized to HumanHT-12 v4 BeadChip Arrays (Illumina Inc, San Diego, CA, USA), and then washed and scanned according to standard Illumina protocols. Data were extracted in GenomeStudio (Illumina) using default analysis settings and no normalization method. Resulting data were imported into GeneSpring GX v11.5 (Agilent Technologies, Santa Clara, CA, USA). Expression values were normalized using quantile normalization with default settings.

## Supplementary Tables and Figure


