# Supplementary material for: Secretome from senescent melanoma engages the STAT3 pathway to favor reprogramming of naive melanoma towards a tumor-initiating cell phenotype
Source: Oncotarget. 2013 Aug 19;4(12):2212–24. doi: 10.18632/oncotarget.1143 (PMC3926821; doi:10.18632/oncotarget.1143)
Supplement: Supplementary file 1 [file oncotarget-04-2212-s001.pdf]

Supplemental Material

Supplementary Figures

Figure S1: The secretome of senescent melanoma cells triggers melanoma initiating-cell phenotype. (A) Immunofluorescence experiments with MITF and E-cadherin antibodies of 501mel cells exposed to secretome of control (Cont) or of senescent (SSMC) melanoma cells. **(B)** Human melanoma cell lines (501mel, WM9, MeWo, G361), exposed to the secretome of control melanoma cells (Cont) or to SSMC were analyzed for MITF content by flow cytometry as described in Fig.1E. **(C)** Cell count of human melanoma cell lines (501mel, WM9) and cells freshly isolated from human biopsy (Patient#1) exposed to secretome of control melanoma cells (Cont) or to SSMC. **(D)** Effect of secretome of control melanoma cells (Cont) or SSMC on 501mel cell viability as illustrated by FACS analysis of violet blue staining. Staurosporine, (1 μM) for 16 hrs, was used as a positive control.

Figure S2: IL6 triggers STAT3 activation. Immunofluorescence experiments with MITF and phospho-STAT3 (p-STAT3) antibodies of 501mel cells exposed or not to IL6 (20 ng/ml).

Figure S3: STAT3 inhibition prevents the decrease in MITF level and the activation of STAT3 mediated by the secretome of senescent melanoma cells. Human melanoma cell lines (501mel, WM9) and cells freshly isolated from human biopsy (Patient#1) were transfected with control or STAT3 siRNA and were exposed to the secretome of control melanoma cells (Cont) or SSMC. Cell lysates were analyzed by western blotting with the indicated antibodies.

Figure S4: Effect of the secretome of senescent melanoma cells on genes implicated in mesenchymal and stemness phenotype. (A) QRT-PCR analysis of MITF, AXL, mesenchymal-related (FN1, CCL2, TGFBI, DKK3, THBS2) and stemness (NANOG, OCT4, ALDH1A3) genes in melanoma cells exposed to SSMC versus control medium. **(B)** Ingenuity pathway analysis (IPA®) was performed on the 52 genes specifically regulated in three melanoma cell types by the SSMC.

Supplementary Tables

Division	Cont,%	SSMC,%
**0**	0,9	1,9
**1**	7,9	26,9
**2**	53,6	57,1
**3**	29,9	11,8
**4**	6,6	2,2

Table S1: Effect of the secretome from control (Cont) or senescent (SSMC) melanoma cells on melanoma populations according to the number of divisions. Quantification by the MACSQuant software.

Division	Cont,%	IL6, %
**0**	0,6	1,4
**1**	5,15	17,9
**2**	38,6	63,9
**3**	43,6	14,1
**4**	11,9	2,7

Table S2: Effect of the secretome from control (Cont) or IL6-treated melanoma cells on melanoma populations according to the number of divisions. Quantification by the MACSQuant software.

Gene symbol	Inhibition (%)	p-value
**HPS4**	42.50	0.0384
**MBP**	41.67	0.0422
**MITF**	28.22	0.0006
**MLANA**	40.26	0.0024
**MLPH**	43.16	0.0481
**OSTM1**	24.02	0.0219
**RAB27A**	39.49	0.0167
**RAB38**	37.45	0.0008
**SLC24A5**	36.28	0.0124

Table S3: Inhibition of MITF and some MITF target genes in 501mel, WM9 and melanoma cells from patient#1 exposed to SSMC. Values were extracted from Illumina BeadChips Arrays.

		**Cont/SSMC, present study**		**Down regulated by MITF, Bertolotto et al, 37**		**Increased in the invasive phenotype, Widmer et al, 36**
**Symbol**	**Entrez Gene Name**	**Log (FC)**	**p-value**	**Log(FC)**	**p-value**	
**DKK3**	dickkopf 3 homolog (Xenopus laevis)	**5.489**	6.39E-03	**-1.5355**	0.0408	**yes**
**LOXL4**	lysyl oxidase-like 4	**3.679**	5.74E-03	**-1.510**	0.0011	**yes**
**ALDH1A3**	aldehyde dehydrogenase 1 family. member A3	**3.495**	4.17E-03	**-2.1818**	0.0006	**yes**
**TIMP3**	TIMP metallopeptidase inhibitor 3	**2.796**	2.36E-03	**-1.8110**	0.0008	**yes**
**PXDN**	peroxidasin homolog (Drosophila)	**2.767**	1.13E-03	**-1.7038**	0.0213	**yes**
**IL1B**	interleukin 1. beta	**2.096**	1.55E-02	**-5.7419**	0.0196	**yes**
**AXL**	AXL receptor tyrosine kinase	**2.072**	4.65E-03	**-1.5008**	0.0022	**yes**
**PMEPA1**	prostate transmembrane protein. androgen induced 1	**1.889**	4.80E-03	**-4.4740**	0.0385	**yes**
**COL8A1**	collagen. type VIII. alpha 1	**1.79**	3.02E-03	**-3.8748**	2.81E-05	**yes**
**TMEM171**	transmembrane protein 171	**1.698**	1.65E-02	**-2.1069**	0.0012	**yes**
**CCL2**	chemokine (C-C motif) ligand 2	**1.686**	2.00E-02	**-4.8434**	0.0404	**yes**
**TMEM47**	transmembrane protein 47	**1.66**	4.74E-03	**-3.3295**	2.74E-05	**yes**
**MYOF**	myoferlin	**1.234**	1.21E-02	**-1.8567**	0.0013	**yes**
**EFEMP2**	EGF containing fibulin-like extracellular matrix protein 2	**1.048**	1.36E-02	**-1.7690**	0.0069	**yes**
**GNG2**	guanine nucleotide binding protein (G protein). gamma 2	**1.025**	4.23E-03	**-1.5772**	0.0266	**yes**

Table S4: Genes that are both up-regulated in melanoma cell lines with invasive phenotype [36] and down-regulated in A375 melanoma cells over-expressing MITF.

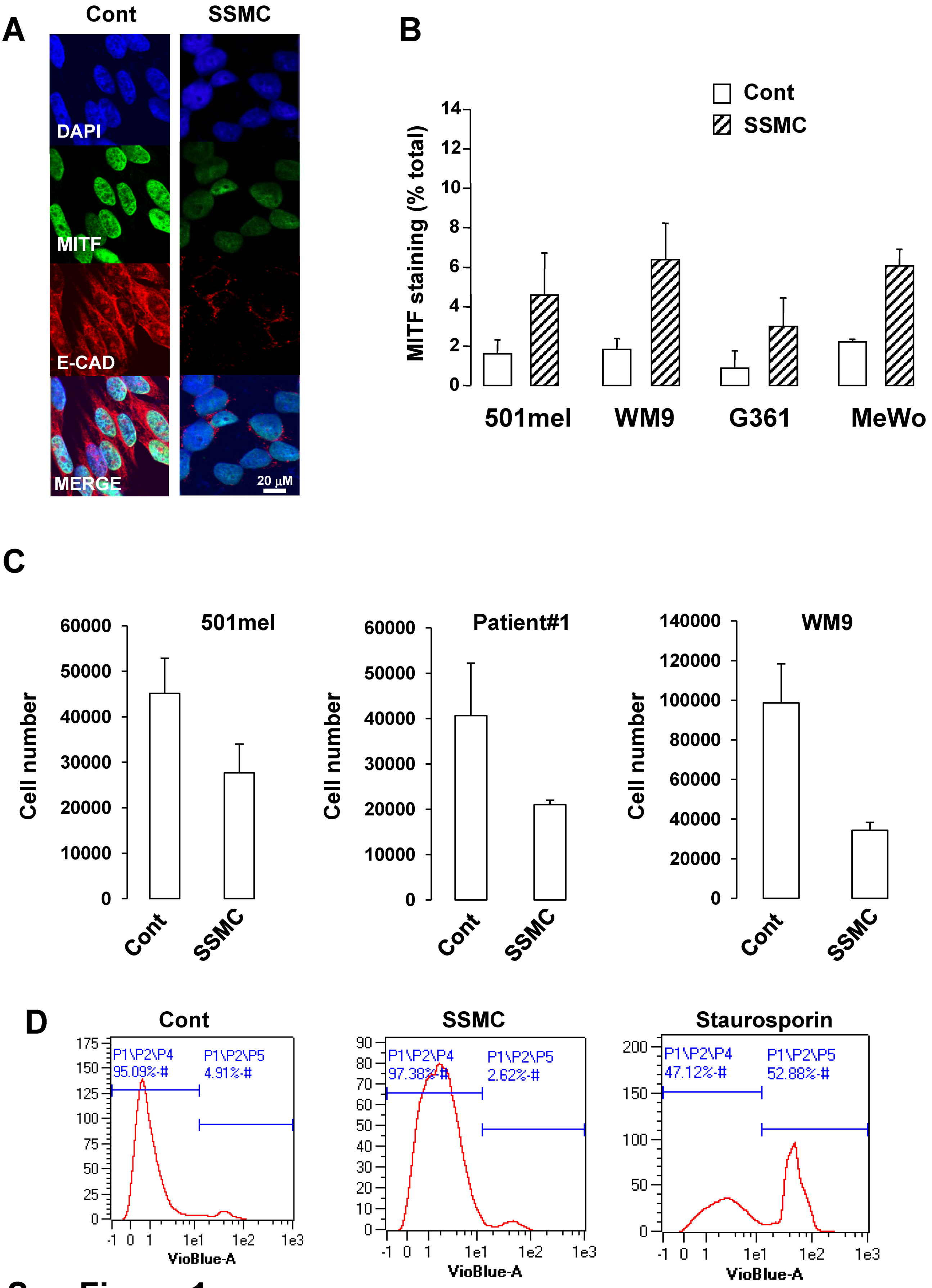

A
Cont
SSMC
DAPI
MITF
E-CAD
MERGE
20 µM
B
Cont
SSMC
MITF staining (% total)
14
12
10
8
6
4
2
0
501mel
WM9
G361
MeWo
C
501mel
Patient#1
WM9
Cell number
Cont
SSMC
D
Cont
SSMC
Staurosporin
P1\P2\P4
95.09%-#
P1\P2\P5
4.91%-#
97.38%-#
2.62%-#
47.12%-#
52.88%-#
VioBlue-A

Sup Figure 1

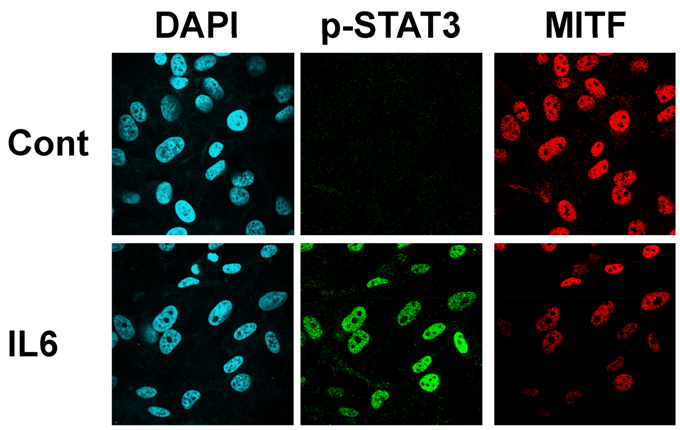

Sup Figure 2

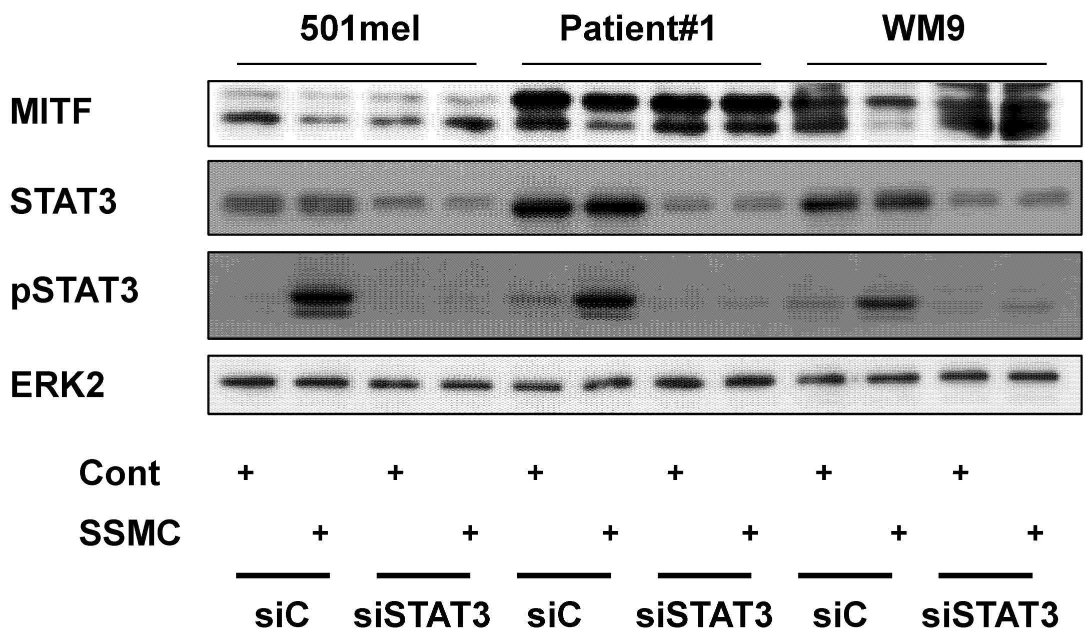
501mel
Patient#1
WM9
MITF
STAT3
pSTAT3
ERK2
Cont
SSMC
+
siC
siSTAT3
siC
siSTAT3
siC
siSTAT3

Sup Figure 3

A

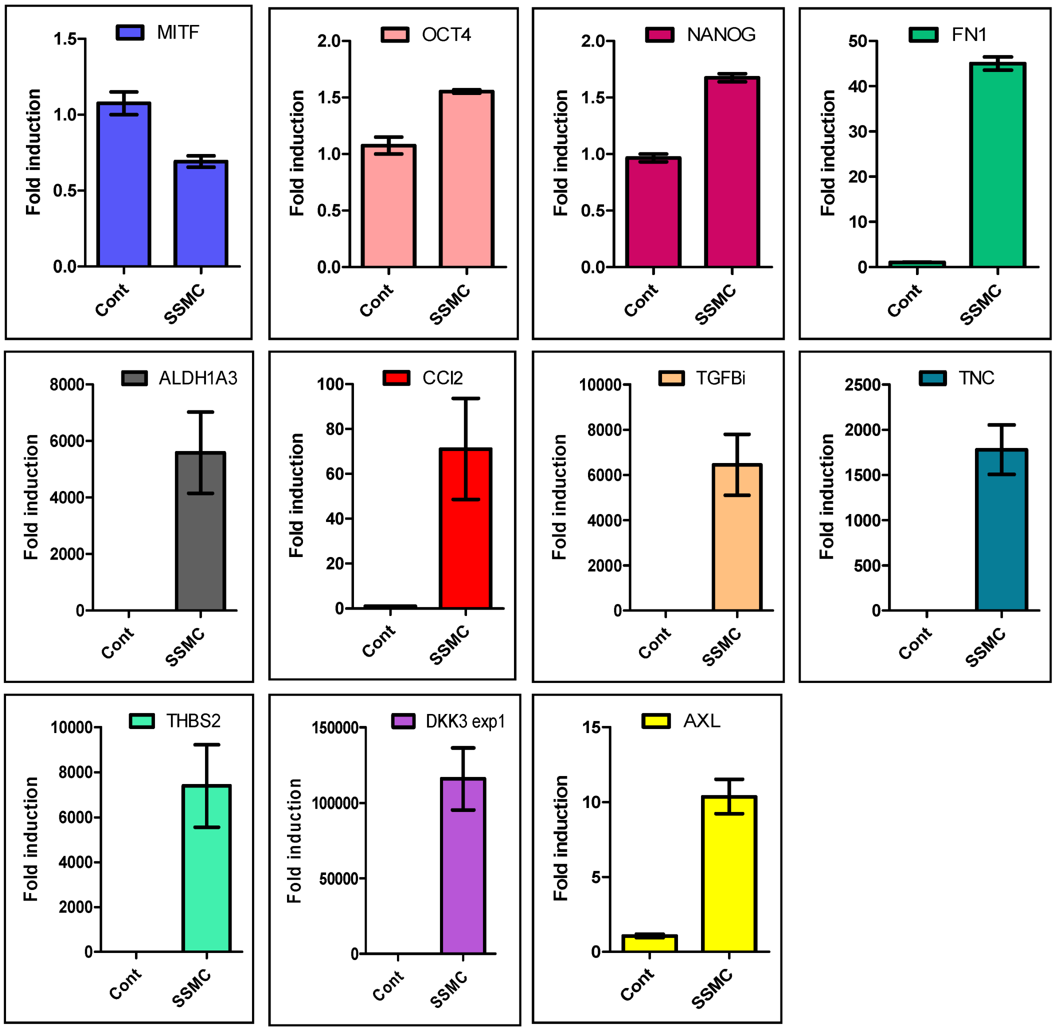

B

Molecular and Cellular Functions

Name	p-value	# Molecules
Cellular Movement	2,78E-07 - 1,35E-02	19
Cell-To-Cell Signaling and Interaction	1,09E-06 - 1,22E-02	16
Cellular Growth and Proliferation	1,95E-06 - 1,22E-02	28
Cellular Assembly and Organization	2,74E-05 - 1,22E-02	22
Cellular Function and Maintenance	2,74E-05 - 1,22E-02	18

Figure S4
